# Psychological Impact of COVID-19 Pandemic on Frontline Responders and Students in Training

**DOI:** 10.1093/geroni/igab046.3722

**Published:** 2021-12-17

**Authors:** Andrew Revell, Mitchell Gauvin

**Affiliations:** 1 University of Massachusetts Dartmouth, University of Massachusetts Dartmouth, Massachusetts, United States; 2 University of Massachusetts Dartmouth, North Dartmouth, Massachusetts, United States

## Abstract

Medical personnel have been in the frontlines of the pandemic leading to increased levels of stress and an impact on mental health. Risks may include, but are not limited to, pronounced burnout (Shechter et al., 2020), vicarious trauma, and post-traumatic stress disorder. The goal of this investigation was to gain insight on the psychological effects that the pandemic had on both frontline responders (EMTs and emergency room staff) and students in clinical training. Emerging adults and adult participants (N=150; ages 18-46; 70.4% ages 18-24) were recruited through the introductory psychology subject pool, community healthcare, and social media. Linear regression and means testing were employed to assess differences between current frontline workers and future workers on the Depression, Anxiety, and Stress Scale (DASS-21; Lovibond, 1995) on irritability, sleep, covid-19 positive presence, concentration, and other mental health factors. Hierarchical linear regression, controlling for age, indicated higher anxiety subscale scores (b=2.49, p=.008) and higher stress subscale scores (b=2.25, p=.035) were present on the DASS-21 for women. Dichotomous means testing indicated higher anxiety, stress, and depression levels for those who also reported a significant change in sleep habits (p <.001) and for those who reported being more irritable on their days off (p <.001) during the pandemic. Students in training (37.7%) indicated interest in considering a different career path (r = .302, p = .02). Future studies should examine these dynamic relationships among mental health factors among healthcare professionals and the implications for training the next generation.

